# Applicability Evaluation of Male-Specific Coliphage-Based Detection Methods for Microbial Contamination Tracking

**DOI:** 10.4014/jmb.2110.10003

**Published:** 2021-10-20

**Authors:** Gyungcheon Kim, Gwoncheol Park, Seohyun Kang, Sanghee Lee, Jiyoung Park, Jina Ha, Kunbawui Park, Minseok Kang, Min Cho, Hakdong Shin

**Affiliations:** 1 Department of Food Science and Biotechnology, College of Life Science, Sejong University, Seoul 05006, Republic of Korea; 2 Food Safety and Processing Research Division, National Institute of Fisheries Science, Busan 46083, Republic of Korea; 3 Division of Biotechnology, SELS Center, College of Environmental and Bioresource Sciences, Jeonbuk National University, Iksan 54596, Republic of Korea

**Keywords:** Male-specific coliphage, double agar overlay method, RT-PCR, virome

## Abstract

Outbreaks of food poisoning due to the consumption of norovirus-contaminated shellfish continue to occur. Male-specific (F+) coliphage has been suggested as an indicator of viral species due to the association with animal and human wastes. Here, we compared two methods, the double agar overlay and the quantitative real-time PCR (RT-PCR)-based method, for evaluating the applicability of F+ coliphage-based detection technique in microbial contamination tracking of shellfish samples. The RT-PCR-based method showed 1.6-39 times higher coliphage PFU values from spiked shellfish samples, in relation to the double agar overlay method. These differences indicated that the RT-PCR-based technique can detect both intact viruses and non-particle-protected viral DNA/RNA, suggesting that the RT-PCR based method could be a more efficient tool for tracking microbial contamination in shellfish. However, the virome information on F+ coliphage-contaminated oyster samples revealed that the high specificity of the RT-PCR- based method has a limitation in microbial contamination tracking due to the genomic diversity of F+ coliphages. Further research on the development of appropriate primer sets for microbial contamination tracking is therefore necessary. This study provides preliminary insight that should be examined in the search for suitable microbial contamination tracking methods to control the sanitation of shellfish and related seawater.

## Introduction 

Shellfish are one of the world’s major vectors for the transmission of enteric viruses including human noroviruses, which cause viral gastroenteritis [1]. Norovirus infection occurs on a steady basis every year, with approximately 200,000 deaths annually worldwide [2]. In Korea, norovirus is a leading cause (18.1%) of food poisoning, with an average of 46 cases and 963.4 patients over the past five years [3]. To prevent viral gastroenteritis, the importance of sanitation in shellfish and related seawater is emphasized [4]. Since the infectious dose of norovirus is at the level of 18-1,000 viral particles, a concentration process is required to detect norovirus in seawater or shellfish, suggesting a limitation of continuous monitoring in the field.

Male-specific (F+) coliphages, which specifically infect coliform bacteria, have been suggested as a promising indicator of fecal contamination. Also, F+ RNA coliphages have been proposed as indicators of the virological quality of seawater since the enteric virus concentrations can be predicted [5-8]. These coliphages are non-pathogenic, stronger than gut bacteria, and last longer in the environment [9]. Screening for these enteroviral markers provides a means to monitor waterborne human viral pathogens. F+ RNA coliphages are RNA-based viruses that are similar in size and shape to noroviruses.

In the United States, F+ RNA coliphages are used as an indicator in the safety management of seawater, and moreover, a standardized method for determining F+ coliphage in soft-shelled clams and American oysters was suggested by the FDA (ISSC Proposal 05-114) based on the double agar overlay method. F+ RNA coliphage has been consistently associated with *Escherichia coli* and is abundantly present in shellfish and seawater of domestic origin. This coliphage has also been proposed as a human enteric virus indicator because of the connection to contamination sources in water-treatment processes and the aquatic environment [10]. Several previous studies have utilized F+ coliphages to determine fecal contamination and its sources [11-13]. In particular, F+ RNA coliphages isolated from human waste were effectively distinguishable from those isolated from other animal waste [14], and contamination sources in water resources [15] and rivers [16] have been identified based on the types of F+ RNA coliphages.

The double agar overlay method is generally used to detect bacteriophages, and it is also used as an F+ coliphage monitoring technique [17-19]. However, the sensitivity of this method for the enumeration of F+ coliphages is affected by various factors. The formation of pili, which is needed for F+ coliphages to attach themselves to host cells and cause infection, is affected by host growth conditions such as temperature [20]. An extremely small amount of coliphage in the sample in combination with interference from somatic phages in the same sample resulted in lowered sensitivity of the plaque assay [21-23]. As an alternative, a highly sensitive and specific real-time PCR (RT-PCR)-based method has been designed for the detection and enumeration of F+ coliphages [17] and enteric viruses in seawater and shellﬁsh [24]. The sensitivity and specificity of quantitative real-time PCR provide advantages over the double agar overlay method. While the RT-PCR-based technique can distinguish subgroups of viruses in samples (F+ DNA coliphage, F+ RNA coliphage, and some subgroups), the double agar overlay method can measure the entire level of F+ coliphages in the samples. In addition, the RT-PCR-based technique has a disadvantage in that it detects both non-particle-protected (naked) DNA and RNA, so it has limitations related to the goal of detecting only the active form of the virus.

Previous studies on the comparison of RT-PCR and the double agar overlay methods for monitoring F+ coliphage in seawater and shellfish were limited. In this study, the characteristics of the RT-PCR-based method and the double agar overlay method were investigated to determine how they can be utilized to monitor F+ coliphage in shellfish samples. In addition, we attempted to support our study by using virome analysis of oyster samples in which coliform was detected.

## Materials and Methods

### Bacterial and Viral Strains and Growth Media

*E. coli* F_amp_ (ATCC 700891) was grown statically at 37°C for 4 h in a broth containing 10 g tryptone, 1 g dextrose, and 5 g NaCl/L of medium [25]. The broth was supplemented with the addition of streptomycin/ampicillin stock (0.1%) after sterilization in an autoclave at 121°C. Bacteriophage MS2 (ATCC 15597; F+ RNA coliphage) was propagated in the *E. coli* growth culture overnight at 37°C. Bacteriophage M13 (ATCC 15669-B1; F+DNA coliphage) was cultured for 30 min, and then kanamycin (25 ug/ml) was added to perform an overnight culture for propagation. The obtained MS2 or M13 bacteriophage culture was centrifuged at 15,000 g for 15 min to separate the host cell debris and the bacteriophage. The supernatant was filtered through a 0.22 um filter and the stock was stored at 4°C [25].

### Artificial Viral Spike Procedure in Seawater and Shellfish Samples

Seawater samples were obtained from a coastal area (Gunsan, Jeonbuk, Korea) and filtered using a mesh prefilter (1 um pore size). Shellfish samples (oyster, conch, sea squirt, scallop, and red shell) were prepared in 10-L tanks of filtered seawater equipped with an aerator and containing a representative number of animals (12 to 15). Reports concerning concentrations of F+ coliphages in surface water showed that F+ coliphage was detected up to a level of about 1.1 to 5.0 PFU/ml in urban rivers [26, 27], and up to 200 PFU/ml in samples where fecal contamination was expected [16]. To apply artificial viral concentrations to seawater samples above normal contamination levels, MS2 (F+RNA) or M13 (F+DNA) phage was spiked to a concentration of 10^5^, 10^4^, and 10^3^ PFU/ml in the prepared water tanks [28]. After an artificial spike for 24 h, the shellfish were shucked and the meats with liquors were placed into sterile test tubes.

### Shellfish Sample Preparation

The weighted soft-shelled clams or oysters were prepared by dividing them into two groups. For the samples of the first group, the growth broth was added at twice the volume of the samples to make a 1:2 (wgt:vol) elution. For the second group of samples, only the midgut gland was prepared, and growth broth was added in the same volume as with the first group. Each sample was homogenized with a blender for 180 s at high speed. Then, 33.0 g of homogenate was immediately weighed and centrifuged for 15 min (15,000 *g*, 37°C). Weighted supernatant was used to determine the levels of F+ coliphage using the double agar overlay method and RT-PCR-based method.

### Double Agar Overlay Method for Determining F+ Coliphage Levels

Procedures for determining the levels of male-specific coliphage in shellfish samples were followed according to a previously described method of the US FDA (ISSC proposal 05-114; [29]). The double agar overlay method contained two layers of agar; a bottom layer with 1.5% agar medium and a top layer with 1.4% soft agar medium. These media contained 10 g tryptone, 1 g dextrose, and 5 g NaCl/L of medium with the addition of agar. Streptomycin sulfate and ampicillin (50 ug/ml each in final) were added to the bottom agar medium. Two hundred microliters of the bacterial host (*E. coli* F_amp_) was added to 2.5 ml of soft agar medium with 2.5 ml of prepared shellfish supernatant, and the mixture was overlaid on 1.5% bottom agar plates. The overlaid plates were incubated at 37°C for 16 h. After incubation, the male-specific (F+) coliphage titer in each shellfish sample was calculated considering the weight of supernatant extracted and the used shellfish samples as previously described [30]. The test was performed in triplicate.

### RT-PCR-Based Method for Determining F+ RNA MS2 Coliphage and F+ DNA M13 Coliphage Levels

The supernatant extracted from each shellfish was filtered through a 0.22 um filter (Millipore, USA). To minimize the effect of the possible PCR inhibitors in shellfish extracts, a growth medium was added to the supernatant in the same volume. The total viral genomes were extracted using a MagListo 5M Forensic Extraction Kit (Bioneer, Korea) according to the manufacturer’s instructions. Real-time qPCR was performed to quantify the level of F+ RNA MS2 coliphage and F+ DNA M13 coliphage in shellfish samples using a CFX96 RT-PCR System (Bio-Rad, USA). To proceed with the amplification of the F+ DNA M13 coliphage genomes, AccuPower GreenStar qPCR Master Mix (Bioneer) was used in the real-time PCR assay. The F+ RNA MS2 coliphage genomes were detected via real-time fluorogenic reverse- transcription PCR using AccuPower GreenStar RT-qPCR Master Mix (Bioneer). RT-PCR was performed in a total volume of 20 μl following the manufacturer’s instructions. Reaction conditions were as follows: 95°C for 10 min, 40 cycles of 95°C for 15 s, 62°C for 60 s, with a final extension at 72°C for 5 min. Amplification was determined to be detectable if the cycle threshold (Ct) value was less than 35 [31, 32].

### Virome Sequencing and Bioinformatics Analysis

Selected extracts of oyster samples were filtered through a 0.22 um filter (Millipore) and concentrated using a protein concentrator PES 3K (Thermo Scientific, USA). Non-particle-protected (naked) DNA and RNA was removed by digestion with a mixture of 50 ul DNase I (5 U/ul) (TaKara, Japan) and 50 ul RNase A (10 mg/ml)(Takara) in 450 ul of concentrates at 37°C for 30 min. The viral nucleic acids were extracted using the QIAamp UltraSens Virus Kit (Qiagen, Germany) according to the manufacturer’s instructions. Thereafter, the purification was performed using the GeneGET Viral DNA/RNA Purification Kit (Thermo Scientific) according to the manufacturer’s instructions.

First-strand cDNA was synthesized using the SuperScript IV First-Strand Synthesis System (Invitrogen, USA) and second-strand cDNA was obtained using the Second Strand cDNA Synthesis Kit (Invitrogen) according to the manufacturer’s instructions. The synthesized double-strand cDNA was then purified with a Wizard SV Gel and PCR Clean-Up System (Promega).

The DNA libraries were constructed using a Nextera-XT DNA Sample Preparation Kit (Illumina, USA) and a Nextera XT Index Kit (Illumina) according to the manufacturer's specifications. The indexed libraries were sequenced on the Illumina MiSeq platform (paired-end 250 bp reads), and sequencing was performed by Sanigen Inc. (Korea). Raw reads were trimmed, and filtered read sequences were assembled *de novo* with CLC Genomic Workbench v12.0 (Qiagen). Taxonomy classification was assigned to assembled contigs with a CLC taxonomy profiling tool against the NCBI viral genome database.

All statistical analyses were performed using R software (V 4.0.3). Each experiment was performed at least in triplicate. Data were expressed as the mean ± standard error of the mean (SEM). Mann-Whitney U test was performed with *p* ≤ 0.05 set as the threshold for significance to compare significant differences among groups.

## Results

### Comparison Between Double Agar Overlay Method and RT-PCR-Based Method

We evaluated the sensitivity for the F+ RNA MS2 coliphage-targeting primer sets presented in previous studies (Table S1). A set of primers targeting the assembly protein-coding gene suggested by O'Connell *et al*. was selected for further detection using the real-time (RT)-PCR-based method (Table S2, Fig. S1) [33]. The RT-PCR technique for detecting F+ DNA M13 phage was also prepared for comparison with the double agar overlay method (Fig. S1).

**Fig. 1:**
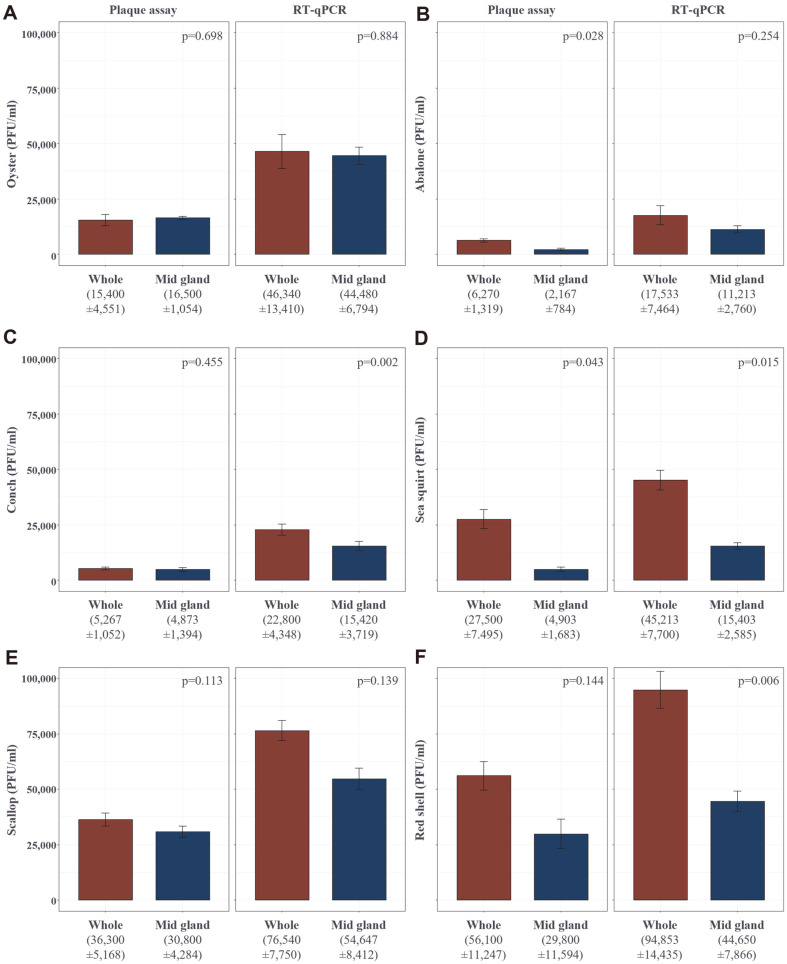
The detection results using the double agar overlay method and real-time PCR-based method for each shellfish artificially infected with F+RNA MS2 for 24 h. (**A**) Oyster (**B**) Abalone (**C**) Conch (**D**) Sea squirt (**E**) Scallop (**F**) Red shell.

To compare the detection efficiency of the double agar overlay method (FDA ISSC Protocol 05-114) and the RT-PCR-based method when F+RNA MS2 coliphage or F+DNA M13 phage were contaminated in shellfish, PFU value was measured after artificially infecting shellfish with coliphages in a water tank. In all 5 types of shellfish (oyster, conch, sea squirt, scallop, and red shell), the RT-PCR-based method showed a higher PFU value than the double-layered method (Fig. 1, Table 1). The RT-PCR method showed 164~432% higher PFU levels in the shellfish samples infected with F+ RNA MS2 phage (*p*-value = 0.170~0.012), in relation to the double agar overlay method, and 534~3,910% higher PFU levels in the shellfish samples infected with F+ DNA M13 phage (*p*-value = 0.008~0.005; Table 1). Since the double agar overlay method can detect only infective phages, these results were derived depending on the proportion of inactivated coliphages in shellfish samples. Also, compared to other shellfishes, the oyster was found to have concentrated most of the F+ coliphage in the mid gland (Fig. 1).

### Comparison of Phage Detection Levels According to Time After the Artificial Spike in Seawater

**Fig. 2:**
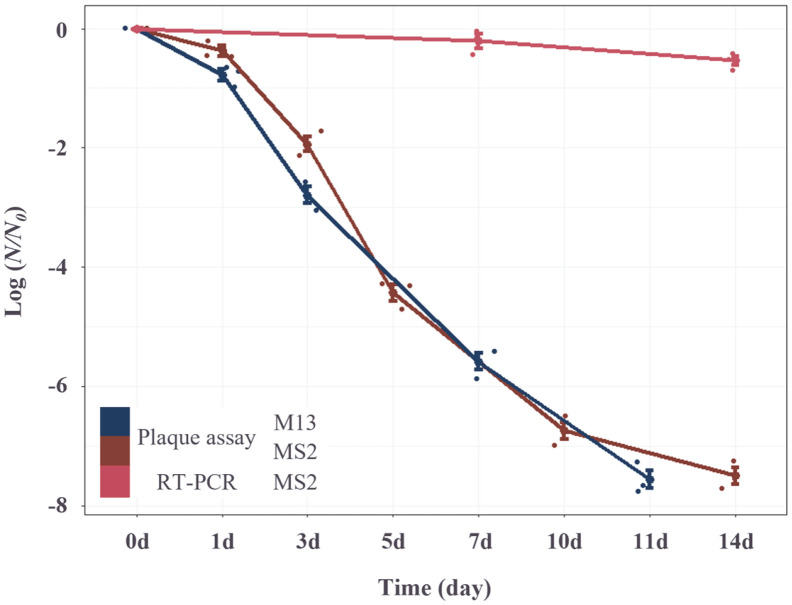
Validation of F+ coliphages detection efficiency according to the time after the spike.

To determine the degree of inactivation of F+ coliphages in seawater with time, the PFU levels for F+ coliphages from artificially spiked seawater was detected by the double agar overlay method and RT-PCR-based method. Both F+ RNA coliphage and F+ DNA coliphage showed a sharp decrease in infective particles from 3 days after the spike, and the PFU values dropped below the detectable range (>10 PFU/ml) after 11 days (Fig. 2). However, the PFU value detected by the RT-PCR method was maintained at a constant level according to the time after the spike, and on day 14, the F+ RNA coliphage showed a 42% decrease and the F+ DNA coliphage decreased by 44%. These results suggest that the double agar overlay method is suitable for detecting infective particles, and the RT-PCR method is suitable for tracking the contamination of indicator coliphage.

### Comparative Analysis of Virome in Oyster according to Detection of F+ Coliphage

**Fig. 3:**
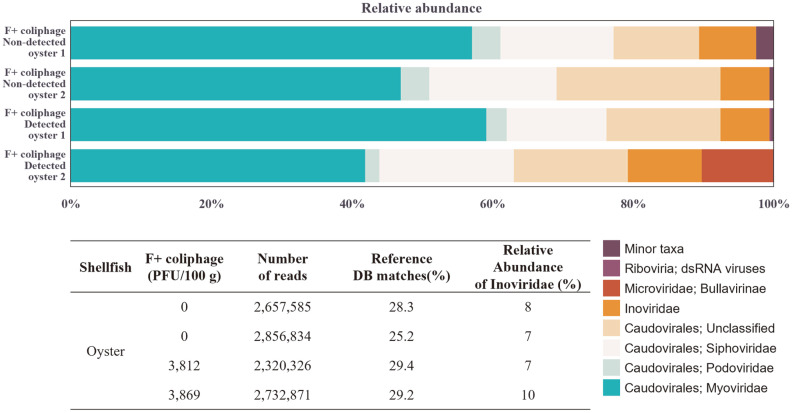
Summary of the virome taxonomic assignments of the oyster samples.

A total of 20 oysters were obtained from a coastal area of Korea, and the infective F+ coliphages were determined using the double agar layer method. F+ coliphages at 3,812 and 3,869 PFU/100 g levels were detected in two samples out of 20 obtained oysters, respectively, and MS2 and M13 coliphages were not detected in all oyster samples by RT-PCR-based method. Virome information was obtained and analyzed for F+ coliphage-positive oyster samples with two negative controls (F+ coliphage non-detected samples). Interestingly, all sequenced samples showed about 7-10% of relative abundance of family *Inoviridae* (F+ DNA coliphage), and there was no correlation between the relative abundance of *Inoviridae* depending on whether infective F+ coliphage was detected or not (Fig. 3). Caudovirales, which includes *Siphoviridae*, *Myoviridae*, and *Podoviridae* families, showed a dominant virome in oyster samples, and no *Fiersviridae* family (F+ RNA coliphage) was identified in any of the samples (Fig. 3). This virome result supports that the PFU values detected by the double agar overlay method show only the level of infective particles.

## Discussion 

This study focused on the appropriateness of the methods (the double agar overlay method and the RT-PCR-based method) for detecting F+ coliphages in shellfish samples. The results support previous reports that quantitative real-time PCR has higher sensitivity and specificity as compared with the classical plaque assay based on the double agar overlay method [17, 34]. We also identified that the RT-PCR method showed 1.6-39 times higher F+ coliphage PFU values from spiked shellfish samples, in relation to the double agar overlay method (Fig. 1, Table 1). This difference is inferred because the RT-PCR-based technique can detect not only an intact virus, but also non-particle-protected DNA, suggesting that the RT-PCR-based method is a more efficient tool for tracking microbial contamination in shellfish. However, the virome results of F+ coliphage-contaminated oyster samples revealed that further research on the development of appropriate primer sets for microbial contamination tracking is necessary. This study provides preliminary insight that should be examined in the search for suitable microbial contamination tracking methods to control the sanitation of shellfish and related seawater.

The differences in sensitivity between the RT-PCR-based method and the double agar overlay method were changed according to the type of shellfish (oyster, conch, sea squirt, scallop, and red shell) and the type of detected coliphage (F+ RNA MS2 coliphage and F+ DNA M13 coliphage). This variation is expected to depend on the effect of shellfish-derived extracts on the complex formation between coliphage and F-pilus of coliform bacteria [35]. This suggests that a technique that restricts shellfish-derived extracts from interacting with F+ coliphage can improve the sensitivity of the double agar overlay method. Since the virome information of this study suggests the diversity of F+ coliphage, further studies are needed to improve the sensitivity of the double agar overlay method targeting multiple F+ coliphages.

Moreover, to verify whether the detection methods in this study can be used for seawater sanitation management in sea areas, the detection level of F+ coliphages (MS2 and M13) in filtered seawater was evaluated for 2 weeks. It was confirmed that the proportion of inactivated F+ coliphages, which could not be detected by the double agar overlay method, increased rapidly with time (Fig. 2), which is thought to be due to the influence of suspended particles of less than 1 um in seawater. This result supports the previous study [6, 36, 37] that the double agar overlay method should be performed within 1 day of harvesting seawater and shellfish samples. In a total of 20 oysters collected in this study, F+ coliphages were detected in 2 oyster samples by the double agar overlay method, but MS2 and M13 coliphages targeted by RT-PCR-based methods were not detected in all samples. These results demonstrated that the RT-PCR-based method shows high sensitivity to the target virus but has limitations when it comes to detecting various coliphages.

F+ RNA MS2 coliphage has physical, chemical, and functional characteristics that are similar to human norovirus, and thus mimic or model human pathogenic viruses. As such, F+ RNA MS2 coliphage is useful in evaluating the sanitation conditions in wastewater treatment processes as well as shellfish harvesting waters. MS2 RNA phage is a member of the family *Leviviridae* (recently renamed as *Fiersviridae*), which is divided into four serogroups, namely, serogroups I to IV [38, 39]. Previous ecological and wastewater studies have revealed that RNA phages from groups II and III are associated with human waste, whereas group I and IV members are predominantly associated with animal waste [14, 39-42]. In this regard, it suggests the possibility of developing a specific primer set for microbial contamination tracking related to human waste. Comparing the genomes of representative coliphages of serogroups I to IV, it can be seen that core gene-coding proteins (Mat, Rep) have more than 20% similarity between serogroups (Fig. S3A). From the results of Mat protein comparative analysis, the possibility of developing specific biomarkers for each serogroup can be inferred (Fig. S3B), and follow-up studies on this will provide key information for the development of microbial contamination tracking methods.

## Supplemental Materials

Supplementary data for this paper are available on-line only at http://jmb.or.kr.

## Figures and Tables

**Table 1:** Comparison of detected PFU values between double agar overlay method and RT-PCR based method.

Contaminated virus in shellfish	PFU values from RT-PCR-based method / PFU values from double agar overlay method (*p*-value)^a^				

	Oyster	Scallop	Conch	Sea squirt	Red shell
*Leviviridae*	300.9% ± 87.1%	210.9% ± 21.4%	432.9% ± 82.6%	164.4% ± 28.0%	169.1% ± 25.7%
(MS2, F+RNA)	(0.062)	(0.024)	(0.012)	(0.170)	(0.029)
*Inoviridae*	968.8% ± 15.4%	716.9% ± 35.0%	3,910.3% ± 449.0%	534.9% ± 3.7%	537.2% ± 11.3%
(M13, F+DNA)	(0.008)	(0.005)	(0.007)	(0.005)	(0.006)

^a^*p*-value was calculated from *t*-test between PFU values derived from RT-PCR-based method and double agar overlay method.
