# Supplementary material for: Applicability Evaluation of Male-Specific Coliphage-Based Detection Methods for Microbial Contamination Tracking
Source: J Microbiol Biotechnol. 2021 Oct 20;31(12):1709–15. doi: 10.4014/jmb.2110.10003 (PMC9705999; doi:10.4014/jmb.2110.10003)
Supplement: Supplementary file 1 [file jmb-31-12-1709-supple.pdf]

Table S1. Primer sets information for real-time PCR assays for detection of bacteriophage MS2

Assay	Start	Primer sequences	Location	Size	Product of target gene	Reference
S1	632	GTCGCGGTAATTGGCGC	Fwd	77 bp	Assembly protein	
	708	GGCCACGTGTTTTGATCGA	Rev			
S2	1155	TGTGGAGAGACAGGGCACTG	Fwd	77 bp	Assembly protein	
	1231	CAGTTGTTGGCCATACGGATT	Rev			
S3	1449	CGTTCACAGGCTTACAAAGTAACCT	Fwd	107 bp	Coat protein	O'Connell et al., 2006
	1555	CCAACAGTCTGGGTTGCCAC	Rev			
S4	1693	CCTCAGCAATCGCAGCAAA	Fwd	115 bp	Lysis protein	
	1807	GGAAGATCAATACATAAAGAGTTGAACTTC	Rev			
S5	2232	GCTCTGAGAGCGGCTCTATTG	Fwd	69 bp	RNA replicase β chain	
	2301	CGTTATAGCGGACCGCGT	Rev			
S6	3165	CGGCTGCTCGCGGATA	Fwd	65 bp	Lysis protein	Kata et al., 2014
	3229	AACTTGCGTTCTCGAGCGAT	Rev			
S7	21	TCCTGCTCAACTTCCTGTCGAG	Fwd	112 bp	none-target	Jolien et al., 2012
	132	CACAGGTCAAACCTCCTAGGAATG	Rev			

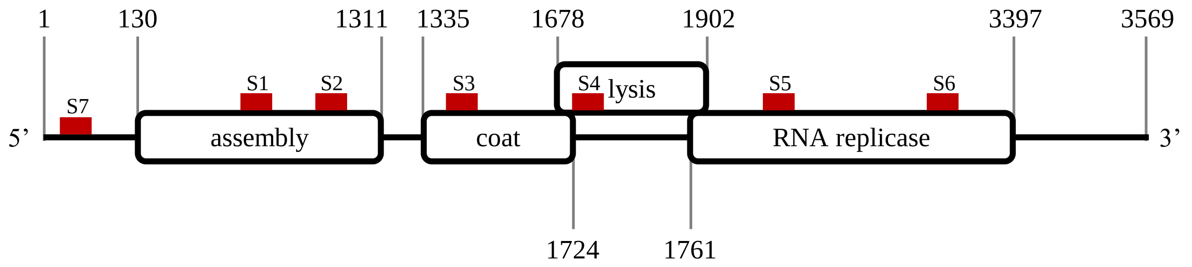

Table S2. Performance of real-time PCR assays of each primer sets targeting bacteriophage MS2

Primer set	7.00E+8	7.00E+7	7.00E+6	7.00E+5	7.00E+4	R^2	Efficiency
Primer 1 (S1)	15.62±0.06	19.23±0.04	23.06±1.17	26.48±0.99	29.15±1.79	0.997	95.70%
Primer 2 (S2)	17.23±0.91	21.10±1.05	23.68±1.17	28.14±1.69	30.69±0.98	0.954	97.06%
Primer 3 (S3)	23.45±1.33	26.68±0.34	30.41±0.25	31.98±0.85	34.72±0.53	0.960	128.79%
Primer 4 (S4)	23.30±016	26.66±0.02	29.55±0.06	33.01±0.06	35.14±0.32	0.994	115.30%
Primer 5 (S5)	25.12±0.10	28.37±0.10	32.31±0.43	34.19±1.33	34.95±0.61	0.920	146.81%
Primer 6 (S6)	17.19±0.03	20.95±0.06	25.05±0.05	28.91±0.20	32.71±0.34	0.999	80.50%
Primer 7 (S7)	22.90±0.13	26.28±0.09	30.44±0.50	33.33±0.35	36.85±1.19	0.988	93.32%

(A)

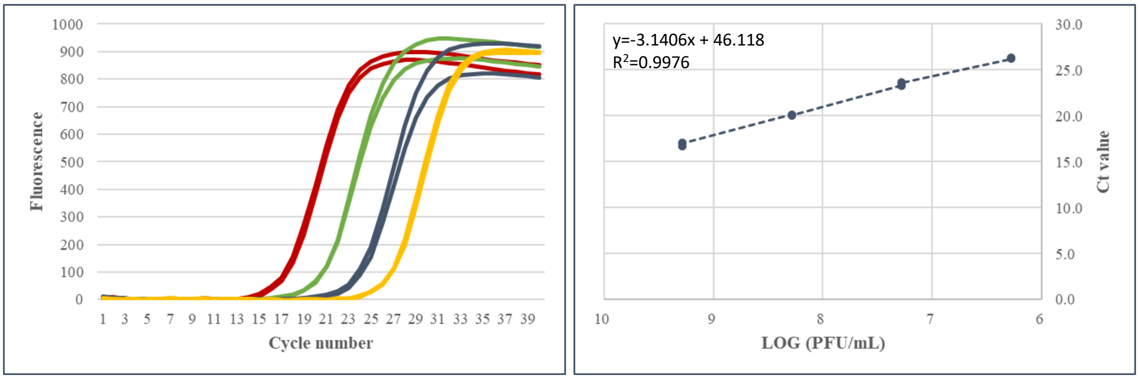

Primer sequences	Location	Size (bp)	Product of target gene	Reference
GTCGCGGTAATTGGCGC	Fwd	77	Assembly protein	O'Connell et al., 2006
GGCCACGTGTTTTGATCGA	Rev			

(B)

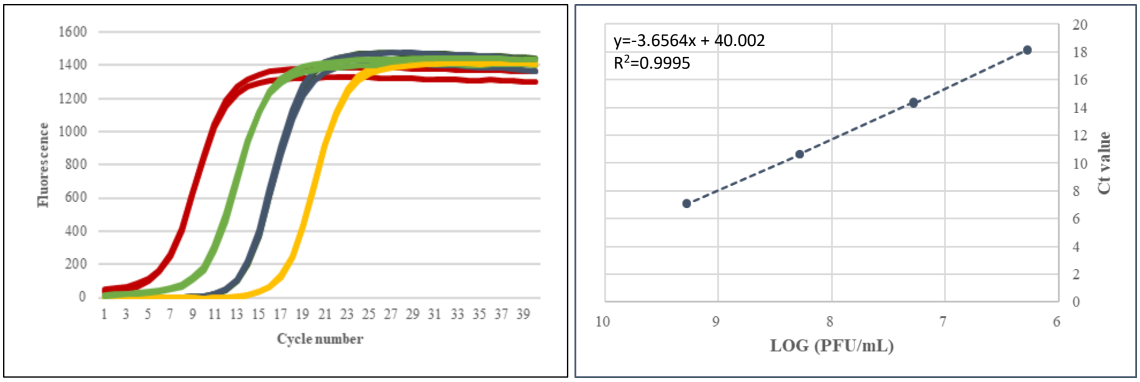

Primer sequences	Location	Size (bp)	Product of target gene	Reference
CACCGTTCATCTGTCCTCTTT	Fwd	96	helix destabilising protein & phage assembly protein	Integrated DNA Technologies
CGACCTGCTCCATGTTACTTA	Rev			

Figure S1. Optimized real-time PCR cycles for standard curve targeting MS2 phage (A) and M13 phage (B)

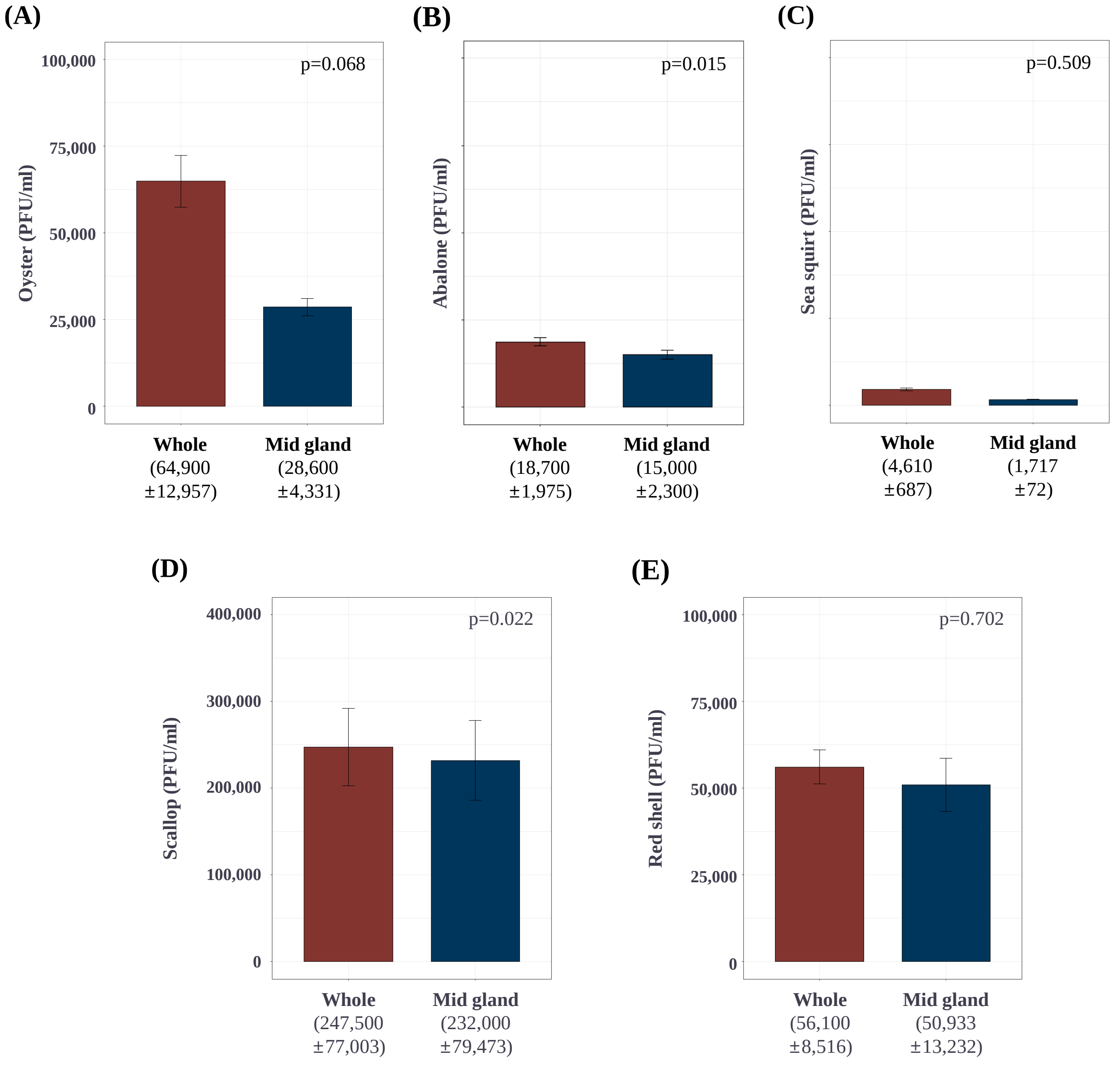

Figure S2. Double layer agar method result for each shellfish artificially infected with F+DNA M13 for 24 hours. (A) Oyster (B) Abalone (C) Seq squirt (E) Scallop (F) Red shell

(A)

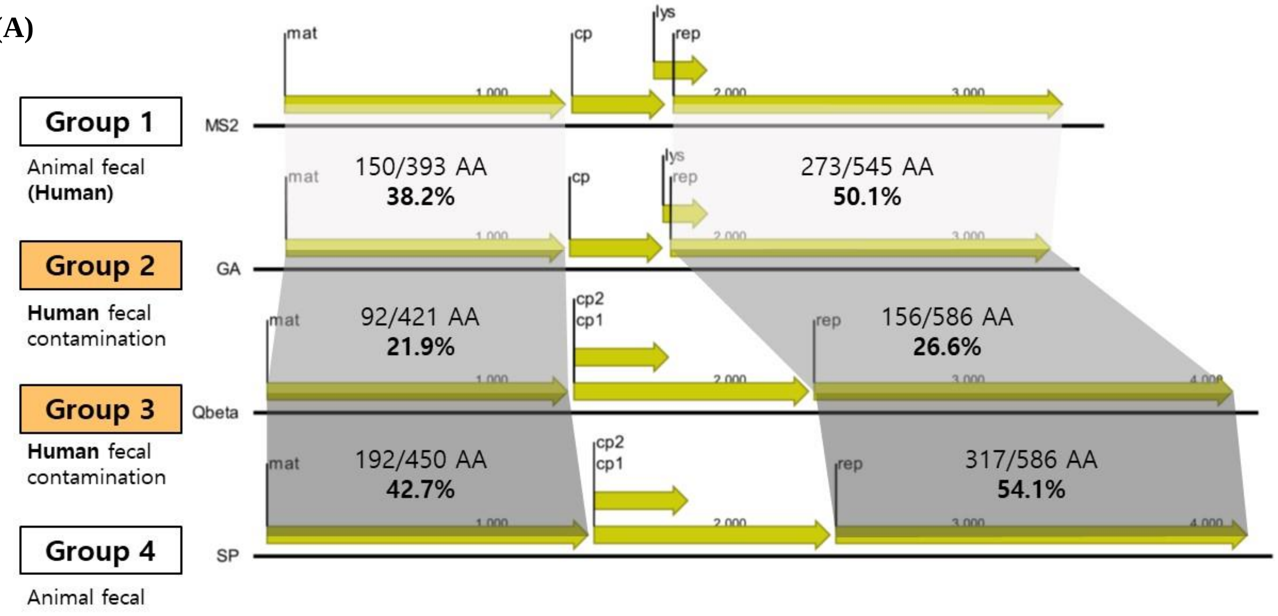

(B)

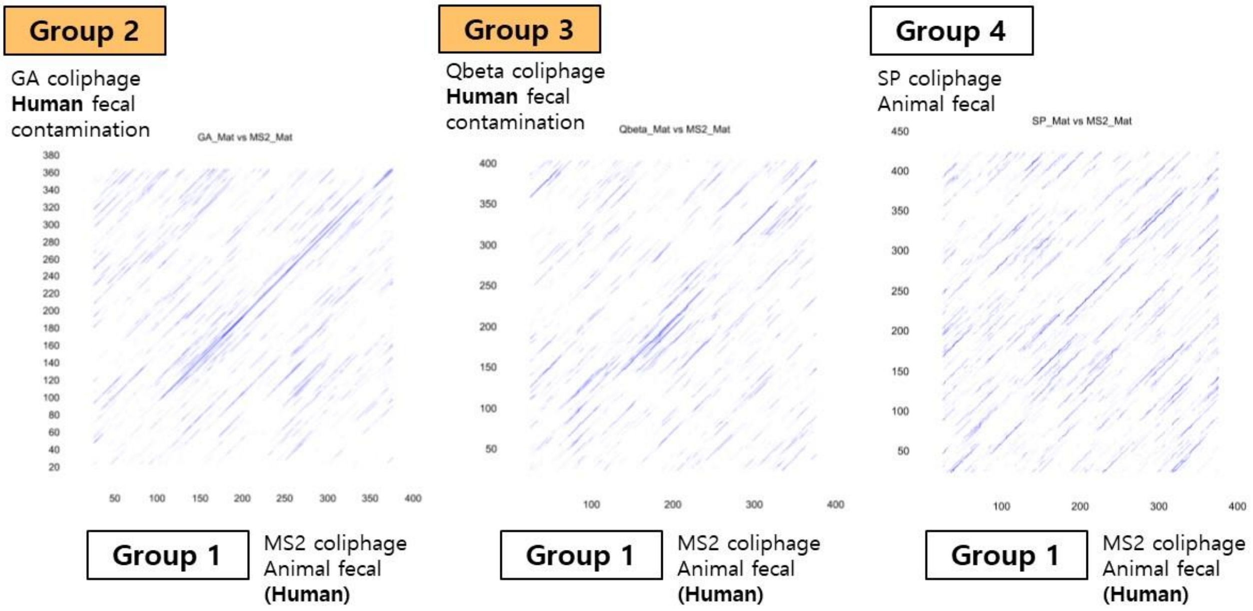

Figure S3. (A) A comparative genomic analysis of representative coliphages in Leviviridae family (B) A comparative Mat protein analysis of representative coliphages in subgroups
